# Xerostomia in patients with advanced cancer: a scoping review of clinical features and complications

**DOI:** 10.1186/s12904-023-01276-4

**Published:** 2023-11-11

**Authors:** Maria Walsh, Norah Fagan, Andrew Davies

**Affiliations:** 1 Marymount University Hospital & Hospice, Curraheen, Ireland; 2 Our Lady’s Hospice & Care Services, Dublin, Ireland; 3 Trinity College Dublin, University College Dublin & Our Lady’s Hospice Dublin, Dublin, Ireland

**Keywords:** Xerostomia, Dry mouth, Neoplasms, Palliative care

## Abstract

**Background:**

The aim of this project was to review the literature on dry mouth / xerostomia in patients with advanced cancer, with the objectives being to determine its prevalence, clinical features, and complications.

**Methods:**

Standard methodology was used to conduct this scoping review. Detailed searches of the Medline, Embase, CINAHL, and PsycInfo databases were conducted to identify relevant studies: eligible studies had to include patients with advanced cancer, and to contain details of clinical features and/or complications of xerostomia. Commercial bibliographic / systematic review software was used to support the process.

**Results:**

Forty-three studies were discovered from the database and hand searches. The studies included 23 generic symptom studies, eight “symptom cluster” studies, nine oral symptom / problem studies, and three xerostomia-specific studies. In depth data is described on the clinical features and complications of xerostomia, and on the “symptom clusters” including xerostomia, in this cohort of patients.

**Conclusion:**

This review discovered a relatively small number of focused studies (involving a similarly small number of patients). Nonetheless, it demonstrates that xerostomia is a very common problem in patients with advanced cancer and is often associated with significant morbidity (and impairment of quality of life).

**Supplementary Information:**

The online version contains supplementary material available at 10.1186/s12904-023-01276-4.

## Background

Xerostomia is defined as “the subjective sensation of dryness of the mouth” [1]. Xerostomia is usually the result of a decrease in the volume of saliva secreted (i.e. resting / unstimulated whole salivary flow rather than stimulated whole salivary flow) [2]. Indeed, normal subjects complain of a dry mouth when their unstimulated whole salivary flow rate falls by 50% [3]. However, xerostomia may also result from a change in the composition of the saliva secreted [4].

Xerostomia is relatively common in the general population. For example, Nederfors et al. [5] estimated an overall prevalence of 21.3% in Swedish adult males, and 27.3% in Swedish adult females: this difference was statistically significant. Other factors associated with xerostomia in this study were age (higher prevalence in older persons), and pharmacotherapy (higher prevalence in persons taking medication, and especially multiple medications). Indeed, medications are the most common cause of xerostomia in the general population [6].

Xerostomia is common in patients with acute / chronic medical conditions [7], and is especially common in patients with cancer. Xerostomia may occur at diagnosis, during anticancer treatment [8], at disease progression, and into cancer survivorship [9]. There are a number of potential causes of xerostomia in patients with cancer, including direct effects of the cancer, indirect effects of the cancer (i.e. paraneoplastic syndrome), adverse effects of anticancer treatments, adverse effects of supportive care measures, and co-morbidities (and their management) [10].

Xerostomia is considered an “orphan symptom” [11], which are defined as “generally prevalent symptoms that are unaddressed in clinical practice, yet often not reported by the patients or by healthcare professionals” [12]. The aim of this review is to appraise the published literature on xerostomia (rather than salivary gland hypofunction) in patients with advanced cancer, with the specific objectives being to determine its prevalence, clinical features (i.e. subjective, objective), and complications (i.e. physical, psycho-social).

## Methods

The methodology utilised in this review was based on the framework developed by Arksey and O’Malley [13], but incorporating updated guidance on this framework [14]. The PRISMA Extension for Scoping Reviews (PRISMA-ScR) was used to report the outcome of this review [15].

### Search strategy

Four electronic databases (Medline, Embase, CINAHL, PsycInfo) were originally searched in October 2022, and re-searched in January 2023 (to check for any new references). A detailed search strategy was developed for Medline (Appendix 1), and adapted as needed for the other databases. Non-English studies were excluded from the review.

### Study eligibility criteria

Studies needed to include patients with advanced cancer, as defined by the National Cancer Institute / NCI, USA [16]: “Cancer that is unlikely to be cured or controlled with treatment. The cancer may have spread from where it first started to nearby tissue, lymph nodes, or distant parts of the body”. Studies which included mixed groups of patients were excluded, unless results for the patients with advanced cancer were separately reported. Studies which focussed on patients with advanced head and neck cancer, and studies that focussed on cancer patients receiving anticancer treatment were excluded. Studies needed to include details of clinical features and/or complications of xerostomia. Studies involving children (< 19 yr) were excluded. Case reports, review articles, and other records without original information were also excluded.

### Data management and synthesis

The EndNote 20™ bibliographic software (Clarivate Analytics LLP, USA) was used to store the retrieved articles, whilst the Covidence systematic review software (Veritas Health Innovation, Australia) was used to screen these retrieved articles.

Two reviewers (MW, NF) independently screened the titles and abstracts for full text articles to review. A third reviewer (AD) was available to resolve potential conflicts. Two reviewers (MW, AD) independently reviewed the full text articles, and extracted the relevant information using a review-specific template. A third reviewer (NF) was again available to resolve conflicts.

The reference lists of all retrieved full text articles, relevant chapters in major palliative care textbooks, and relevant sections of major palliative care guidelines, were hand searched for other potential studies. Other sources of studies included the researchers’ personal bibliographies.

## Results

### Search results

**Fig. 1:**
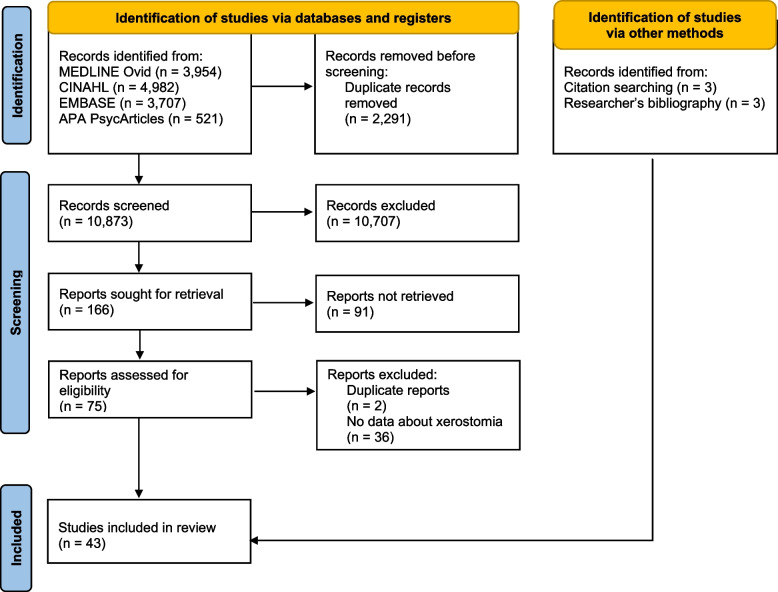
PRISMA flow diagram

The search strategy identified 10,873 references, although only 166 full text articles were retrieved (Fig. 1). Thirty-seven studies were identified from the database searches and had their data extracted [2, 17–52]. Another six studies were identified from handsearching / researcher’s bibliography [53–58]. The studies identified included 23 generic symptom studies [18, 20–22, 25, 26, 29–32, 40–47, 52–56], eight symptom cluster studies [34–39, 57, 58], nine oral symptom / problem studies [17, 19, 23, 24, 27, 28, 33, 50, 51], and three xerostomia-specific studies [2, 48, 49]. Several “duplicate” records were identified amongst the retrieved full text articles: some were conference abstracts, some articles reporting “early” results, and some articles reporting different analyses / subsets of results. Table 1 shows studies reporting clinical features of xerostomia in patients with advanced cancer, and includes references for relevant assessment tools [59–61].

**Table 1:** Studies reporting clinical features of xerostomia in patients with advanced cancer

STUDY	STUDY POPULATION	METHODOLOGY	STUDY RESULTS
Davies et al., 2021 [17]	*n* = 250 Mixed cancer: gastrointestinal (32%), lung (18%), breast (14%) Median age: 68 yr (range 36–91 yr) Female: 58%	Oral Symptom Assessment Scale (OSAS) Frequency: options—“rarely”, “occasionally”, “frequently”, “almost constantly” Intensity: options—“slight”, “moderate”, “severe”, "very severe" Distress: options—“not at all”, “a little bit”, “somewhat”, “quite a bit”, “very much”	Prevalence: 83.5% Most common oral symptom reported Frequency: “rarely”- 9%, “occasionally” - 18.5%, “frequently” - 39%, “almost constantly”- 33.5% Intensity: “slight”- 23%, “moderate”- 41%, “severe"- 26.5%, "very severe"- 9.5% Distress: “not at all”- 18%, “a little bit”- 30%, “somewhat”- 21%, “quite a bit”- 19%, “very much”- 12%
Webber et al., 2021 [18]	*n* = 1507 Mixed cancer: gastrointestinal (52%), urological (12%), lung (10%) Median age: 66 yr (no range) Female: 48%	Memorial Symptom Assessment Scale-Short form (MSAS-SF) [59] Distress: options—“not at all”, “a little bit”, “somewhat”, “quite a bit”, “very much”	Prevalence: 70% 4^th^ most common symptom reported Distress: “not at all/a little”- 40%, “somewhat”- 20%, “quite a bit/very much”- 40%
Tebidze et al., 2019 [19]	*n* = 50 Mixed cancer: lung (37%), urological (13%), breast (11%), gynaecological (11%) Age range: 20–75 yr Female: 40%	Non-validated questionnaire Intensity: options—“slight”, “moderate”, “severe”	Prevalence: 74% Intensity: “slight”- 19%, “moderate”- 46%, “severe”- 35%
Vigstad et al., 2019 [20]	*n* = 274 Mixed cancer: gastrointestinal (29.5%), lung (25%), urological (15.5%) Median age: 69 yr (range 38–90 yr) Female: 49%	Edmonton Symptom Assessment Scale (ESAS)—Norwegian version Intensity: 0–10 NRS; 0 = “none”, 1–3 = “mild”, 4–6 = “moderate”, 7–10 = “pronounced”	Prevalence: 73% Intensity: “none” - 27%, “mild” - 20%, “moderate” - 24%, “pronounced” - 29%
Van Lancker et al., 2017 [21]	*n* = 400 Mixed cancer: lung (23%), gynaecological (12%), gastrointestinal (10%) Mean age: 75 yr (range 65–93 yr) Female: 48%	Assessment Symptoms Palliative Elderly (ASPE) [60]. [ASPE appears to have been modified in this study] Frequency: options—“rarely”, “sometimes”, “often”, “always” “Intensity”: options—“not”, “somewhat”, “moderate”, “very serious”	Prevalence: 77% Most common symptom reported Frequency: “rarely”- 2%, “sometimes”- 33%, “often”- 32%, “always”- 33% Intensity: “not” - 25%, “somewhat”- 13%, “moderate”- 25.5%, “very serious”- 36.5%
Alsirafy, 2016 [22]	*n* = 89 Mixed cancer: gastrointestinal (26%), central nervous system (21%), lung (14%) Median age: 53 yr (no range) Female: 42%	Open questioning about symptoms / non-validated questionnaire Intensity: options—“mild”, “moderate”, “severe”	Prevalence: 57% (questionnaire) 6^th^ most common symptom reported One patient reported xerostomia on open questioning Intensity: “mild”- 57%, “moderate”- 27.5%, “severe”- 15.5%
Mercadante et al., 2015 [23]	*n* = 669 Mixed cancer: gastrointestinal (38%), lung (21%), haematological (10.5%) Mean age: 72.1 yr (SD ± 12.3 yr) Female: 49%	Non-validated questionnaire Intensity: 0–10 NRS; 0 = “absence of symptom”, 10 = “maximum intensity a patient can imagine”	Prevalence: 40.4% Intensity: mean 5.4 (SD ± 2.1)
Fischer et al., 2014 [24]	*n* = 104 “Terminally ill cancer patients” Mean age: 66 yr (SD ± 16.3 yr) Female: 59%	Non-validated questionnaire Frequency: 0–10 NRS Intensity: 0–10 NRS; 0 = “no dry mouth”, 10 = “dry mouth as much as can be”	Prevalence: 91% Frequency: mean 5.8 (SD ± 2.5) Intensity: mean 5.02 (SD ± 3.07); median 6.06
Oechsle et al., 2013 [25]	*n* = 40 Mixed cancer: lung (30%), gastrointestinal (15%), gynaecological (15%) Median age: 63 yr (range 37–88 yr) Female: 55%	Memorial Symptom Assessment Scale—German version (modified) Frequency: options—“rarely” (score = 1), “occasionally” (score = 2), “frequently” (score = 3), “almost constantly” (score = 4) Intensity: options—“slight” (score = 1), “moderate” (score = 2), “severe” (score = 3), "very severe" (score = 4) Distress: options—“not at all” (score = 0), “a little bit” (score = 1), “somewhat” (score = 2), “quite a bit” (score = 3), “very much” (score = 4)	Prevalence: 63% Frequency: mean 1.55 (SD ± 1.50) Intensity: mean 1.42 (SD ± 1.39) Distress: mean 1.07 (SD ± 1.40)
Al-Shahri et al., 2012 [26]	*n* = 124 Mixed cancer: breast (27.4%), head and neck (15.3%), gastrointestinal (12.9%) Median age: 56 yr (range 20–92 yr) Female: 59%	Non-validated questionnaire Intensity: 0–10 NRS; 0 = “absence”, 10 = “greatest severity”	Prevalence: 69.4% Intensity: mean 4.5 (SD ± 2.3) Intensity: median 5 (range 0–10)
Alt-Epping et al., 2012 [27]	*n* = 101 Mixed cancer: gastrointestinal (30%), lung (22%) breast (14%) Age: < 60 yr—39.5%, ≥ 60 yr—60.5% Female: 59.5%	Non-validated questionnaire Intensity: 0–10 NRS; 0–1 = “quite low”, 4–5 = “moderate”, 9–10 = “quite high”	Prevalence: 82% Intensity: 0–1 - 37.5%, 2–3 - 7%, 4–5 - 29%, 6–8 - 6%, 9–10 - 20.5%
Wilberg et al., 2012 [28]	*n* = 99 Mixed cancer population: gastrointestinal (21%), lung (19%), prostate (11%) Mean age: 63.9 yr (SD ± 12.3 yr) Female: 53.5%	Edmonton Symptom Assessment Scale—Norwegian version Intensity: 0–10 NRS; higher scores = higher intensity	Prevalence: 78% Intensity: mean 4.7 (SD ± 3.0)
Spichiger et al., 2011 [53]	*n* = 103 Mixed cancer: urological (23.5%), gastrointestinal (18.5%), lung (16.5%) Mean age: 63 yr (range 19–89 yr) Female: 38%	Memorial Symptom Assessment Scale (MSAS) [61] Frequency: options–“rarely” (score = 1), “occasionally” (score = 2), “frequently” (score = 3), “almost constantly” (score = 4) Intensity: options—“slight” (score = 1), “moderate” (score = 2), “severe” (score = 3), "very severe" (score = 4) Distress: options—“not at all” (score = 0), “a little bit” (score = 1), “somewhat” (score = 2), “quite a bit” (score = 3), “very much” (score = 4)	Prevalence: 65% (admission) Frequency: mean 2.66 Intensity: mean 2.44 Distress: mean 2.03
Webber et al., 2011 [54]	*n* = 120 Mixed cancer diagnosis: gastrointestinal (28%), breast (14%), lung (13%), urological (13%) Median age: 61 yr (range 20–87 yr) Female: 54%	MSAS-SF (see above)	Prevalence: 70% Distress: “not at all”/ “a little bit”- 44%, “somewhat”- 19%, “quite a bit” / “very much”- 37%
Kirkova et al., 2010 [55]	*n* = 181 Mixed cancer: gastrointestinal (29.5%), lung (24%), haematological (9.5%) Mean age: 64 yr (SD ± 13 yr) Sex: no data	Non-validated questionnaire Distress: options—“bothersome / distressful”, “not”	Prevalence: 66% Distress: “bothersome / distressful” - 50%
Bovio et al., 2009 [29]	*n* = 143 Mixed cancer: lung (36.5%), gastrointestinal (33.5%), urological (7%) Mean age: 68 yr (range 57–79 yr) Female: 35%	Non-validated questionnaire (adapted from MSAS-SF) [Xerostomia deemed present if patient was distressed “somewhat”/ “quite a bit”/ “very much”, but not “a little bit”]	Prevalence: 73% Xerostomia associated with anorexia (*p* = 0.012), and dysphagia for solids (*p* = 0.032) Xerostomia associated with reduced energy intake (*p* = 0.006)
McMillan et al., 2009 [30]	*n* = 275 Mixed cancer: lung (33%), gastrointestinal (25.5%), urological (12.5%) Mean age: 72.7 yr (SD ± 11.7 yr) Female: 45.5%	MSAS—modified version Intensity: options—“slight” (score = 1), “moderate” (score = 2), “severe” (score = 3), "very severe" (score = 4) Distress: options—“not at all” (score = 0), “a little bit” (score = 1), “somewhat” (score = 2), “quite a bit” (score = 3), “very much” (score = 4)	Prevalence: 71.3% 3^rd^ most common symptom reported Intensity: mean 2.2 (SD ± 1.1) Distress: mean 1.7 (SD ± 1.3) Higher distress score associated with higher score on Clinical Epidemiological Scale – Depression (*p* = 0.003)
Tsai et al., 2006 [31]	*n* = 77 Mixed cancer: gastrointestinal (44%), lung (23.5%), gynaecological (10.5%) Median age: 62 yr (range 16–86 yr) Female: 61%	Non-validated questionnaire Intensity: options–“none” (score = 0), “mild” (score = 1), “moderate” (score = 2), “severe” (score = 3),	Prevalence: 53.9% (admission) Intensity (admission): mean 0.7 (SD ± 0.8)
Tranmer et al., 2003 [56]	*n* = 66 Mixed cancer: “metastatic cancer or stage IV lymphoma” Mean age: 64.14 yr (SD ± 12.16 yr) Female: 56%	MSAS (see above)	Prevalence: 82% 2^nd^ most common symptom reported Frequency: “frequently” / “almost constantly”- 76% Severity: “moderate” / “severe” / “very severe”- 91% Distress: “quite a bit” / “very much”- 37%
McMillan et al., 2002 [32]	*n* = 178 “Patients with cancer newly admitted to hospice home care” Mean age: 71 yr (range 37-95 yr) Female: 40%	MSAS—modified version Distress: options—“not at all” (score = 0), “a little bit” (score = 1), “somewhat” (score = 2), “quite a bit” (score = 3), “very much” (score = 4)	Prevalence: 78% 3^rd^ most common symptom reported Distress: mean 2.5 (SD ± 1.1)
Davies et al., 2001 [2]	*n* = 120 Mixed cancer: “most common cancer diagnoses were carcinoma of breast, bronchus, prostate, and large bowel” Median age: 66 yr (range 19–89 yr) Female: 61%	MSAS (see above) Additional questions about “mouth discomfort”, “difficulty chewing”, and “difficulty speaking”	Prevalence: 78% 4^th^ most common symptom reported Frequency: “rarely” - 4.5%; “occasionally” - 20.5%; “frequently” - 39.5%; “almost constantly” - 35.5% Severity: “slight” - 14%; “moderate” - 36.5%; “severe” - 33.5%; “very severe” - 16% Distress caused: “not at all” - 16%; “a little bit” - 21.5%; “somewhat” - 22,5%; “quite a bit” - 26%; “very much” - 14% Severity of xerostomia correlated with severity of mouth discomfort (*p* < 0.001); difficulty speaking (*p* < 0.001); “change in the way food tastes” (*p* = 0.001); lack of appetite (*p* = 0.005); difficulty chewing (*p* = 0.01); difficulty swallowing (*p* = 0.01)
Oneschuk et al., 2000 [33]	*n* = 99 Mixed cancer: lung (28%), gastrointestinal (27%), genitourinary (18%), Mean age: 67 yr (SD ± 12.7 yr) Female: 58%	Non-validated questionnaire Intensity: 0–10 NRS Relative “importance” versus other symptoms / problems: options–“not important” (score = 1), “slight importance” (score = 2), “some importance” (score = 3), “moderate importance” (score = 4), “considerable importance” (score = 5), “very important” (score = 6), “great importance” (score = 7)	Prevalence: 88% Intensity: mean 6.2 (SD ± 2.21) Relative importance: mean 3.6 (SD ± 1.67)

### Assessment

The three xerostomia specific studies involved small numbers of patients (median: 70; range: 16–120) [2, 48, 49]: two were quantitative (with one using a validated / non-specific assessment tool, i.e. Memorial Symptom Assessment Scale / MSAS) [2, 48], whilst one was qualitative [49]. The nine oral symptom / problem studies involved somewhat larger numbers of patients (median: 104; range: 50–669) [17, 19, 23, 24, 27, 28, 33, 50, 51]: all were quantitative (with three using validated / non-specific assessment tools, i.e. Oral Symptom Assessment Scale / OSAS, Edmonton Symptom Assessment System / ESAS—Norwegian version, and MSAS) [17, 28, 50]. It should be noted that there is no validated xerostomia assessment tool for this cohort of patients.

### Epidemiology

Xerostomia prevalence varied widely in the studies identified in this review (median: 72.15%, range: 40.4–91.0%). Alsirafy et al. reported that only one patient reported this symptom on open questioning, although 57% patients gave a positive response on systematic assessment (with 43% of these patients reporting “moderate” / “severe” intensity) [22]. Other authors reported similar findings in this group of patients [62].

The identified studies reported minimal information on the risk factors for xerostomia (e.g. demographics, cancer diagnosis, performance status, comorbidities). There is some data to suggest that xerostomia may be more prevalent in females [20, 40], in younger patients [41], and in Caucasians versus African Americans in this population [42].

Xerostomia appears to be common in all groups of patients with cancer, including patients with haematological malignancies [23, 43], and patients with sarcomas [44]. There is better data to suggest that xerostomia is more prevalent in patients with a poor performance status [45, 46], and equally that xerostomia is more prevalent in patients at the very end-of-life [31, 47]. However, the association between xerostomia and limited prognosis is inconsistent [63].

The identified studies also reported minimal information on the aetiology of xerostomia. Davies et al. (2001) reported 97.5% patients were receiving medications that are known to cause xerostomia, and that the median number of such drugs used was 4 (range 0–9) [2]. Other authors have reported an association with the use of anticholinergic drugs [41], opioid analgesics [41], and chemotherapy drugs [23].

### Symptom clusters

Table 2 shows studies reporting physical and/or psychological symptom clusters involving xerostomia [34–39, 57, 58]. The symptom clusters identified varied from study to study, and also varied within study (depending on the outcome measure chosen, and the statistical method utilised). It should be noted that there are many other studies reporting physical and/or psychological symptom clusters in patients with advanced cancer, but which did not include the symptom of xerostomia [64].

**Table 2:**
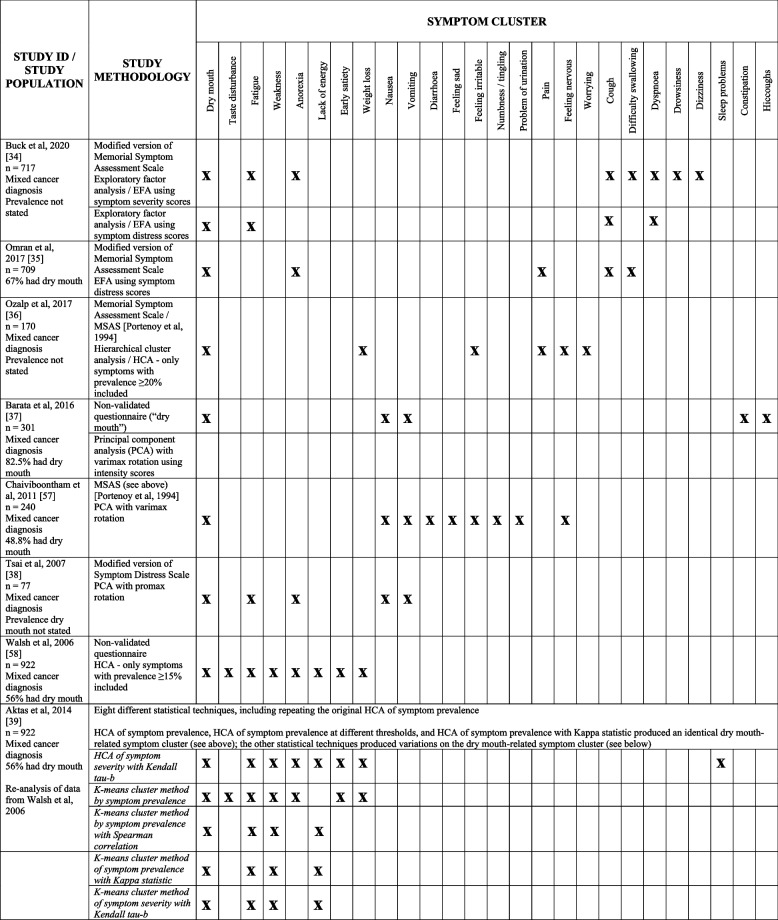
Studies reporting symptom clusters including dry mouth in patients with advanced cancer

Davies et al. (2021) examined oral symptom clusters, and reported that xerostomia did not cluster with other oral symptoms when using prevalence data, but did cluster with taste disturbance when using frequency data (Spearman’s rank correlation coefficient = 0.6) [17]. No analogous studies were identified in the literature.

### Clinical features

Table 1 shows studies reporting the clinical features of xerostomia. It demonstrates that it is usually a frequent symptom [2, 17, 21, 56], is often moderate-to-severe in intensity [2, 17, 19–22, 27, 56], and is often associated with significant distress [2, 17, 18, 54–56]. Moreover, xerostomia is a usually a continuous symptom [41], occurring both during the day-time and during the night-time (often resulting in sleep disturbance) [48, 49]. It should be noted that there were many other studies reporting xerostomia in patients with advanced cancer, but which did not include details about clinical features and/or complications.

### Complications

Xerostomia has been associated with a variety of other oral symptoms / problems, including oral discomfort [2, 28], difficulty opening mouth (“gluing” of mouth) [49], taste disturbance [2, 17, 49], difficulty chewing [2], difficulty swallowing [2, 29, 49], and difficulty speaking [2, 49]. Oral discomfort may result from the xerostomia itself, and/or the complications of the xerostomia (e.g. dental erosion leading to dental sensitivity, and possibly trauma to the oral mucosa) [10].

As well as the complications already outlined, xerostomia is a common “nutrition impact symptom” [65], and has been associated with anorexia [2, 29], decreased enjoyment of eating [49], a need to take longer while eating, and a need to drink more while eating. Unsurprisingly, xerostomia is associated with decreased food / energy intake [29]. Furthermore, patients with xerostomia often avoid eating with others (“social eating”) [49].

Xerostomia has been associated with non-specific oral infections [49], but especially with oral candidosis [27, 50, 51]. Importantly, it is also associated with periodontal disease and dental caries, which can rapidly progress to cause problems such as oral discomfort / pain, halitosis, tooth loss, local infections, and systemic infections [10]. Xerostomia has also been associated with problems relating to the absorption / efficacy of oral transmucosal medications [66]. Unsurprisingly, given all of the above, xerostomia is associated with social isolation (self-imposed) [49], decreased mood / depression [30, 49], decreased spiritual well-being [52], and reduced quality-of-life.

## Discussion

This scoping review confirms that xerostomia is a very common problem, and is frequently associated with significant morbidity (and impaired quality of life), in patients with advanced cancer. Indeed, this review reiterates that this so-called “orphan symptom” warrants much greater appreciation from healthcare professionals. Thus, patients with advanced cancer should be regularly screened for xerostomia, and those with xerostomia require adequate assessment, appropriate treatment, and ongoing re-assessment (the so-called “ART” of management) [67].

Saliva has a variety of functions (e.g. oral lubrication, mucosal protection, oral hygiene, infection control, communication, eating and drinking), and many of the reported oral symptoms / problems relate to these homeostatic functions. However, patients also experience indirect problems, especially psychosocial complications (e.g. depression, social isolation). Indeed, xerostomia could be considered an “orphan syndrome” as opposed to an orphan symptom. Importantly, while most problems are associated with increased morbidity, some problems may be associated with increased mortality (e.g. oral infections causing systemic infections; nutrition-related symptoms exacerbating malnutrition).

The management of xerostomia involves treatment of the cause (if possible), use of saliva stimulants (e.g. chewing gum, muscarinic agonists), use of saliva substitutes (e.g. water, “artificial salivas”), and/or treatment of any complications [10, 67]. Saliva substitutes are very different from normal saliva, and so tend to have minimal effect on the related oral symptoms / problems. Moreover, they tend to have a limited effect on the sensation of dryness of the mouth. Hence, expert opinion recommends the use of saliva stimulants wherever possible [67], since an increase in secretion of “normal” saliva should improve both the sensation of dryness of the mouth and the related oral symptoms / problems. In addition, the use of appropriately fluoridated toothpastes (or mouthwashes) is recommended to prevent dental caries in dentate patients with xerostomia / salivary gland hypofunction [10].

In terms of future research, further observational studies of xerostomia are probably unnecessary in patients with advanced cancer (given the available evidence). However, further interventional studies are very necessary, since relevant evidence is lacking, especially in this cohort of patients [67]. Future studies need to assess not only improvement in the sensation of dryness of the mouth, but also improvement in the related oral symptoms / problems (and especially those associated with significant morbidity / increased mortality).

## Conclusion

This scoping review discovered a relatively small number of focused studies (involving a similarly small number of patients). Nonetheless, it demonstrates that xerostomia is a very common problem in patients with advanced cancer and is often associated with significant morbidity (and impairment of quality of life).

### Supplementary Information

**Additional file 1: Appendix 1.** Medline search strategy.

## Data Availability

Not applicable. No datasets were generated or analysed during the current project.

## Abbreviations

NCI: National Cancer Institute
MSAS: Memorial Symptom Assessment Scale
OSAS: Oral Symptom Assessment Scale
ESAS: Edmonton Symptom Assessment System
ART: Assessment, re-assessment, treatment
