# Supplementary material for: Xerostomia in patients with advanced cancer: a scoping review of clinical features and complications
Source: BMC Palliat Care. 2023 Nov 11;22:178. doi: 10.1186/s12904-023-01276-4 (PMC10638744; doi:10.1186/s12904-023-01276-4)
Supplement: Supplementary file 1 — Additional file 1: Appendix 1. Medline search strategy. [file 12904_2023_1276_MOESM1_ESM.docx]

**APPENDIX 1 – MEDLINE SEARCH STRATEGY**

1. Dry mouth – key word
2. Xerostomia – MeSH term
3. Salivary gland hypofunction – key word
4. Salivary gland dysfunction – key word
5. Oral symptoms – key word
6. Oral problems – key word
7. Oral health – MeSH term
8. Oral care – key word
9. Mouth care – key word
10. Mouth diseases – MeSH term
11. Oral diseases – key word
12. *1 or 2 or 3 or 4 or 5 or 6 or 7 or 8 or 9 or 10 or 11*
13. Neoplasms – MeSH term
14. Cancer – key word
15. *13 or 14*
16. Palliative care – MeSH term
17. Terminal care – MeSH term
18. End of life care – key word
19. *16 or 17 or 18*
20. *12 and 15*
21. *12 and 19*
22. *21 not 20*
23. *20 or 22*
